# Comparative effectiveness, safety and persistence of ocrelizumab versus natalizumab in multiple sclerosis: A real-world, multi-center, propensity score-matched study

**DOI:** 10.1016/j.neurot.2025.e00537

**Published:** 2025-01-28

**Authors:** Elena Barbuti, Alessia Castiello, Valeria Pozzilli, Antonio Carotenuto, Ilaria Tomasso, Marcello Moccia, Serena Ruggieri, Giovanna Borriello, Roberta Lanzillo, Vincenzo Brescia Morra, Carlo Pozzilli, Maria Petracca

**Affiliations:** a Department of Human Neurosciences, Sapienza University, Rome, Italy; b Department of Neurosciences, Reproductive Science and Odontostomatology, University of Naples "Federico II", Naples, Italy; c Unit of Neurology, Neurophysiology and Neurobiology, Campus Bio-Medico University, Rome, Italy; d Department of Molecular Medicine and Medical Biotechnology, University of Naples "Federico II", Naples, Italy; e Department of Neurosciences, San Camillo-Forlanini Hospital, Rome, Italy; f MS Center, San Pietro Fatebenefratelli Hospital, Rome, Italy; g Department of Public Health- University of Naples "Federico II", Naples, Italy

**Keywords:** Multiple sclerosis, Natalizumab, Ocrelizumab, Real-world, Effectiveness, Safety

## Abstract

Ocrelizumab (OCR) and Natalizumab (NTZ) are highly effective treatments widely used in Multiple Sclerosis (MS). However, long-term, real-world comparative data on clinical effectiveness, safety and treatment persistence are limited. This retrospective analysis included relapsing and progressive MS patients initiating treatment at two Italian Universities (“La Sapienza” and “Federico II”). Propensity-score nearest-neighbor matching with a caliper of 0.1 was conducted to adjust for between-group differences in age, sex, previous treatment status, MS phenotype, disease duration, clinical and MRI activity at baseline. Differences in follow-up duration were adjusted with pairwise censoring. Cox proportional hazard regression models were used with Evidence of disease activity (EDA-3) and its components (relapses, MRI activity, and confirmed disability progression) as outcomes. Treatment discontinuation rate and occurrence of adverse events (AEs) were tested using logistic regression. We identified 308 patients (140 on OCR, 168 on NTZ) with a mean (SD) follow-up of 75.7 (30.8) months. Patients treated with OCR were older and less active and less frequenlty naïve at baseline than NTZ-treated patients. The PS-matching procedure retained 140 patients (70 pairs) with a mean follow-up of 55.9 (14.3) months. No significant differences were found between NTZ and OCR in terms of relapses, MRI activity or confirmed disability progression. OCR treatment was associated with a higher incidence of mild to moderate AEs, and higher to comparable treatment persistence. This study provides real-world evidence of comparable effectiveness between OCR and NTZ over a 5-year observation period, with OCR being associated with a higher incidence of AEs and, possibly, higher treatment persistence.

## Introduction

Multiple sclerosis (MS) is a chronic inflammatory demyelinating disease of the Central Nervous System (CNS). The therapeutic scenario of the disease has changed in the last 20 years thanks to the availability of a wide range of Disease Modifying Treatments (DMTs) with different characteristics and mechanisms of action. Several studies demonstrated the benefit of initiating high-efficacy therapy (HET) from disease onset, rather than lower efficacy treatment with subsequent escalation in case of residual inflammatory activity [[1], [2], [3], [4]]. Early intervention with HET is thought to mitigate neuroinflammation, which is more pronounced in the early stages of MS, thus preventing the accumulation of irreversible injury and subsequent clinical outcomes [5,6]. Among HET, there is still no consensus on the choice of one drug over another in clinical practice, except in the case of contraindications due to comorbidities or safety issues. Monoclonal antibodies are ranked as high-efficacy therapies in meta-analyses comparing treatment efficacy from Randomized Clinical Trials (RCTs) [[7], [8], [9]]. Natalizumab and Ocrelizumab are high-efficacy monoclonal antibodies widely used in clinical practice. Natalizumab (NTZ) is a humanized IgG4 monoclonal antibody targeting α4 integrin subunit of the α4β1 and α4β7 leukocyte adhesion molecules, thus preventing their interaction with vascular cell adhesion molecule 1 (VCAM-1) and Mucosal vascular addressin cell adhesion molecule 1 (MAdCAM-1) on endothelial cells [10]. This mechanism of action inhibits lymphocyte migration across the blood-brain barrier into the CNS. In Europe, NTZ it is indicated in monotherapy in adults with highly active relapsing-remitting MS (RRMS) for patients not responding to a full and adequate course of treatment with at least one DMT or with rapidly evolving severe RRMS (defined by at least two disabling relapses in one year and at least one Gadolinium enhancing lesion on brain MRI, or a significant T2 lesion load increase compared to a previous MRI). In the U.S., NTZ is indicated as monotherapy for the treatment of patients with relapsing forms of MS, including clinically isolated syndrome, relapsing-remitting disease, and active secondary progressive disease. Ocrelizumab (OCR) is a second-generation humanized monoclonal antibody targeting CD20, a cell surface antigen found on pre-B cells, mature and memory B-cells. B lymphocytes produce proinflammatory cytokines and antibodies, and activate proinflammatory T cells through antigen presentation, thus playing a pivotal role in MS pathogenesis [11]. Ocrelizumab is indicated for the treatment of adult patients with relapsing multiple sclerosis (RMS) or early (age <55 years; disease duration <10 years, and EDSS ​≤ ​6.5) and active (MRI showing inflammatory activity as new T2 lesions in comparison to a previous MRI or Gd-enhancing lesions) primary progressive multiple sclerosis (PPMS). So far, real-world effectiveness comparative studies between NTZ and OCR mostly focused on relapsing-remitting MS (RRMS) and had a relatively short follow-up, ranging from 1 to 3 years. Here we extended previous findings evaluating differences in efficacy, safety and persistence of NTZ-vs OCR-treated patients with RRMS and PMS over a medium-term follow-up.

## Materials and methods

### Study design and population

We retrospectively analyzed data from MS patients who started treatment with NTZ or OCR in two Italian tertiary centers: S.Andrea Hospital, Rome and AOU “Federico II”, Naples. Clinical and MRI data were collected prospectively by each treating center and retrospectively retrieved for this study. Data retrieval took place in March 2024 and focused on patients starting NTZ or OCR for clinical practice or as part of open-label studies (LIBERTO and ENSEMBLE) or RCTs (OPERA and WA21493) and then followed for clinical practice, with a minimum on-treatment persistence of 2 years. All patients were diagnosed according to McDonald criteria applicable at the time of diagnosis [12,13]. Patients starting NTZ or OCR as first treatment (naïve) as well as patients switching to NTZ or OCR from other DMTs (switchers) were included, with the exception of patients switching from NTZ to OCR or vice versa.

A study-specific database was created to harmonize data collection from the two centers. For each patient the following characteristics were retrieved: sex, date of birth, date of MS onset, MS phenotype, prior DMTs’ history, date of first and last infusion of NTZ or OCR, date and reason for NTZ or OCR discontinuation, presence of disease activity (relapses and/or MRI activity as defined below) in the 2 years preceding NTZ or OCR start, Expanded Disability Status Scale (EDSS) score at baseline and over the follow-up (until last available visit), occurrence and dates of relapses and MRI activity during follow-up (until last available visit), presence and date of adverse events (AEs), and, if present, date and reason for switching to other DMTs during follow-up.

### Outcome measures

#### Effectiveness

We considered NEDA-3 status and its components (absence of clinical relapses, MRI activity, and sustained disability worsening) as the primary outcome [14]. A relapse was defined as the appearance of acute neurological symptoms, for at least 24 ​h in the absence of infection or change in body temperature, separated by at least 30 days from onset of a preceding clinical demyelinating event [15]. MRI activity was defined as new T2 hyperintense lesions compared with a previous scan and/or Gd-enhancing lesions. Disability worsening was defined as an increase of 1.5, 1 or 0.5 points from a baseline EDSS of respectively 0, <5.5 or =/>5.5, confirmed after 6 months [15]. Disability worsening was further sub-categorized in relapse-associated worsening (RAW) and progression independent of relapse activity (PIRA). PIRA was defined as a disability worsening event that occurred more than 90 days after, or more than 30 days before, the onset of a relapse. RAW was defined as a disability worsening from the study baseline occurring within 90 days following the onset of a relapse or up to 30 days prior to it [16].

#### Safety

We considered the development of AEs as primary safety outcome. Adverse events were classified according to Common Terminology Criteria for Adverse Events (CTCAE) v5.0 (Publish Date: November 27, 2017) [17]. AEs were graded as Grade 1-mild (no treatment required and no interference with daily living activities), Grade 2-moderate (may require treatment and cause some interference with functioning), Grade 3- severe (systemic drug or other treatment required, interruption of daily living activities), Grade 4-life-threatening (immediate risk of death) [18] and Grade 5 Death related to AE.

#### Persistence

Discontinuation from treatment during follow-up was reported as outcome of choice.

### Statistical analysis

Statistical analyses were performed in R 4.3.1 and Jamovi 2.4.12.0. Categorical variables were presented as numbers or percentages while continuous variables were presented as mean (standard deviation, sd) or median (interquartile range, IQR). Normality of variables was tested using the Kolmogorov-Smirnov test. Nonparametric or parametric tests were used according to the normality distribution of data. Unpaired 2-tailed T-test, X^2^ and Mann-Whitney *U* test were used as appropriate to assess differences in demographic variables. Two tailed p values lower than 0.05 were considered significant.

Using the R package “MatchIt” we conducted a 1:1 propensity-score (PS) nearest-neighbor matching, with distance ​= ​glm and a caliper of 0.1 to adjust for between-group differences in age, sex, previous treatment status (naïve vs previously treated with DMTs), clinical and MRI activity at baseline, phenotype (RRMS, SPMS, PPMS) and disease duration. Logistic regressions were used to estimate individual PS values with the previously cited baseline characteristics as covariates and treatment group as the dependent variable. The validity of PS-based matching was tested by analysis of mean standardized differences (|d|), with |d| ​< ​0.05 considered a good balance. Pairwise censoring was used to adjust for differences in the length of follow-up between the 2 groups, right-censoring at the shorter individual follow-up treatment length within each pair. Cox proportional hazard regression models were used to compare matched groups. The presence of any disease activity (the counterpart of NEDA-3) and of its subcomponents was entered as the primary and secondary outcomes. Relapses within 2 months from OCR and 3 months from NTZ start were not included in the analysis, as they could be due to rebound activity in patients switching from previous DMTs and not necessarily reflect a lack of effectiveness of current treatment [19,20]. For the same reason, MRI activity within 4 weeks of treatment was excluded for both DMTs [19,20]. Also, MRI activity occurring within 1 month from a clinical relapse was considered associated with the clinical relapse and therefore excluded from the MRI activity model.

Treatment discontinuation rate and occurrence of AEs were tested via logistic regression in matched cohorts and Odd Ratios (OR) were reported.

### Ethics statement

This study complied with the ethical standards of the 1964 Declaration of Helsinki and its amendments. Due to its retrospective design, it did not affect patient care, and specific ethical approval was not required as all assessments were part of routine clinical practice at specialized centers. In line with Italian regulations, each site's principal investigator notified the local ethics committee. Patients provided informed consent for clinical data collection.

## Results

### Study population

**Fig. 1:**
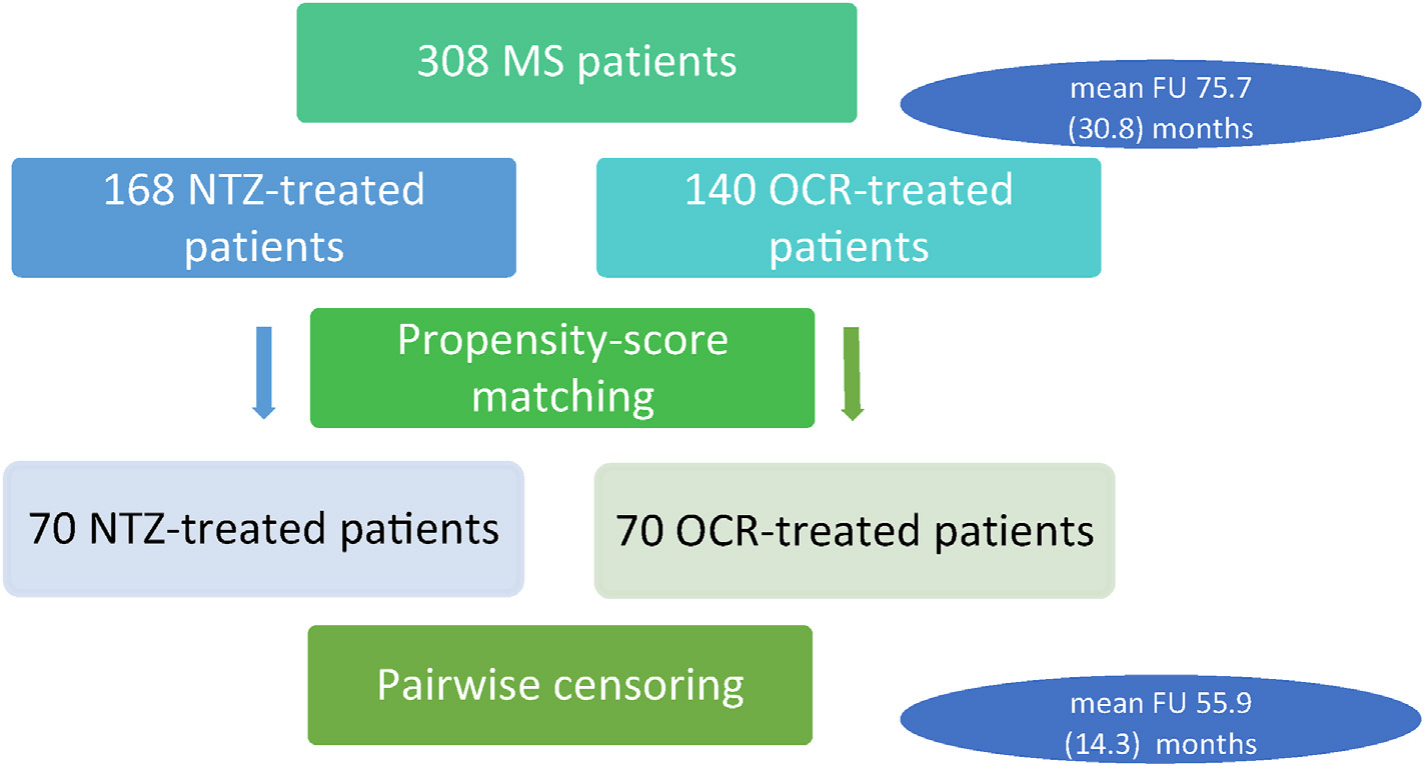
**Study flow-chart.** FU: follow-up, MS: multiple sclerosis, NTZ: natalizumab, OCR: ocrelizumab.

308 patients (168 on NTZ, 140 on OCR), with a mean (standard deviation) follow-up of 75.7 (30.8) months were eligible for analysis [Fig. 1]. Demographic and clinical characteristics of eligible patients are reported in Table 1. Patients treated with OCR were older, less active at baseline, less frequently naive than NTZ-treated patients and showed higher neurological disability (p ​< ​0.05, see Table 1 for details). One-hundred and forty patients (70 pairs) were retained after PS-matching. No difference in baseline clinical and demographic data was present in the matched cohort (p ​> ​0.05 for all, see Table 1 for details). As treatment duration was longer in NTZ than in OCR-treated patients (mean (sd): 77.8(28.8) vs 71.2(32.8); p ​= ​0.045), pairwise censoring was applied before any other analysis to avoid bias due to differences in follow-up. After the procedure, mean follow-up time was 60 months (55.9 ± 14.3) for both groups.

**Table 1:** Demographic and clinical characteristics of the total cohort and of the matched cohort.

	ORIGINAL COHORT			MATCHED COHORT		
	Natalizumab(168)	Ocrelizumab(140)	p	Natalizumab(70)	Ocrelizumab(70)	p
Age (years), mean (sd)	42 (11)	49.9 (11)	**<0.001**^a^	44.7 (11.1)	45.1 (11)	0.813^a^
Female sex, n(%)	121 (72)	85 (60.71)	**0.036**^b^	45 (64.28)	47 (67.14)	0.722^b^
Disease duration (years) mean (sd)	15.7 (8.04)	15.9 (8.18)	0.806^c^	14.6 (8.01)	14.4 (7.68)	0.98^c^
Naïve patients, n(%)	57 (33.9)	32 (22.86)	**0.033**^b^	21 (30)	23 (32.86)	0.716^b^
Active patients at baseline^d^, n(%)	147 (87.5)	59 (42.14)	**<0.001**^b^	52 (74.29)	51 (72.86)	0.848^b^
Phenotype at DMT start			**<0.001**^b^			0.829^b^
RRMS	152	57		56	55	
SPMS	13	47		11	13	
PPMS	3	36		3	2	
EDSS at baseline, median (IQR)	3 (0–6.5)	4.5 (0–7.5)	**<0.001**^c^	3 (0–6.5)	2.75 (0–7.5)	0.924^c^
Patients treated with previous DMT, n						
Injectables^e^	99	96		41	36	
Orals^f^	44	88		23	36	
Intravenous^g^	3	10		0	5	
Treatment duration, (months), mean (sd)	85.2 (32.2)	64.3 (24.7)	**<0.001**^c^	77.8 (28.8)	71.2 (32.8)	**0.045**^c^

Significant p values (p < 0.05) are provided in bold. DMT: disease modifying treatment, EDSS: expanded disability status scale, IQR: interquartile range, sd: standard deviation.

a t-test for independent values.

b X^2^.

c Mann-Whitney.

d Active patients were defined as having either at least a relapse or MRI activity (new T2 lesions or T1 gadolinium-enhancing lesions) in the 2 years preceding treatment start.

e Interferons (Interferon-Beta 1a or 1b subcutaneous or intramuscular: Extavia, Rebif, Avonex, Betaferon, Plegridy) or Glatiramer acetate.

f Dimethyl fumarate, Teriflunomide, Fingolimod and other S1P modulators, azathioprine.

g Mitoxantrone, Daclizumab, Alemtuzumab, Rituximab.

### Effectiveness analysis

**Fig. 2:**
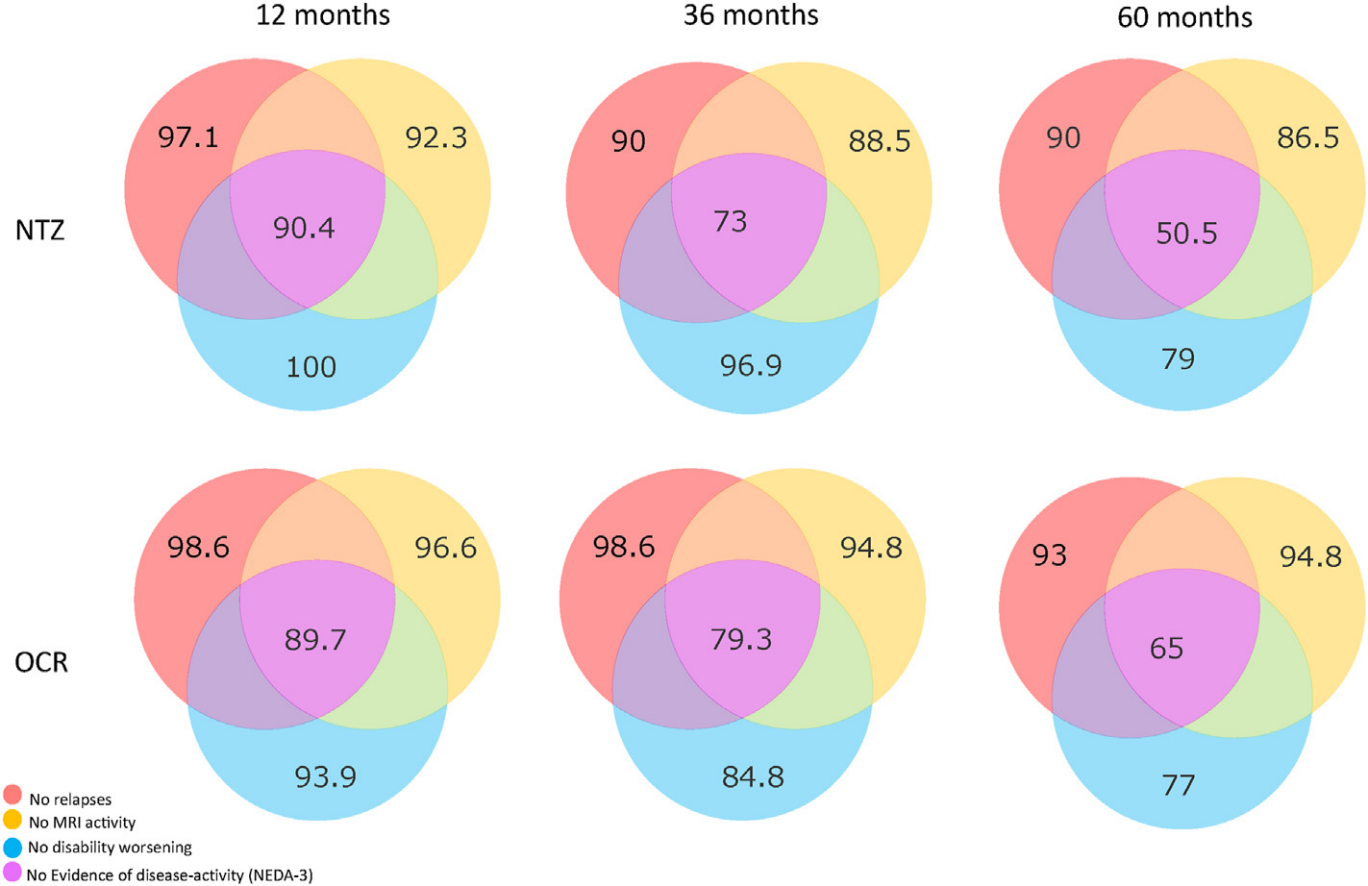
Probability of being free from relapses, MRI activity, EDSS progression and to be NEDA-3 at 12,36 and 60 months.

At 5-year follow-up, the proportions of patients reaching NEDA-3 were 50.5 ​% in NTZ group and 65 ​% in OCR group ([HR 0.64 (0.34–1.24), p ​= ​0.187].) (Fig. 2).

Out of 140 patients, we observed 7 relapses in the NTZ group and 3 relapses in the OCR group. One relapse occurred within a month from NTZ start and was not considered in the statistical analysis. The proportion of relapses did not differ between NTZ- and OCR-treated patients [HR 0.41 (0.11–1.57), p ​= ​0.193].

MRI activity (data available for 52 patients treated with NTZ and 58 treated with OCR) did not differ between NTZ- and OCR-treated patients [HR 0.37 (0.10–1.44), p ​= ​0.152]. 7 patients treated with NTZ experienced MRI activity and 3 patients treated with OCR experienced MRI activity.

Neurological disability at both baseline and follow-up was evaluated in 66 NTZ- and 66 OCR-treated patients: 9 and 12 patients presented EDSS progression over the follow-up in NTZ and OCR group, respectively. No differences were found between the 2 groups [HR 1.43 (0.60–3.40), p ​= ​0.417]. In our cohort no progression was classified as RAW. All patients undergoing confirmed disability progression experienced PIRA.

**Fig. 3:**
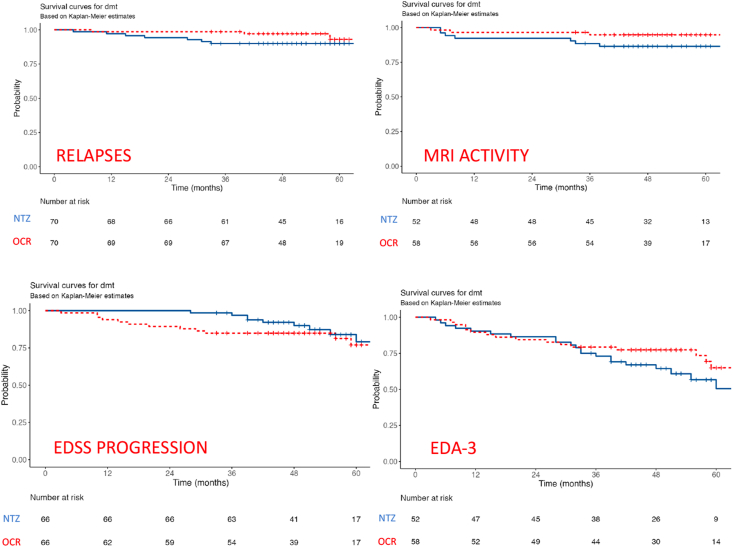
Survival plots based on Kaplan-Meier estimates for time to first relapse, MRI activity, EDSS progression and evidence of disease activity.

Survival plots are shown in Fig. 3. Probabilities to be free of relapses, MRI-activity, EDSS progression or to be NEDA-3 at 1,3 and 5 years are shown in Fig. 2.

### Safety analysis

**Fig. 4:**
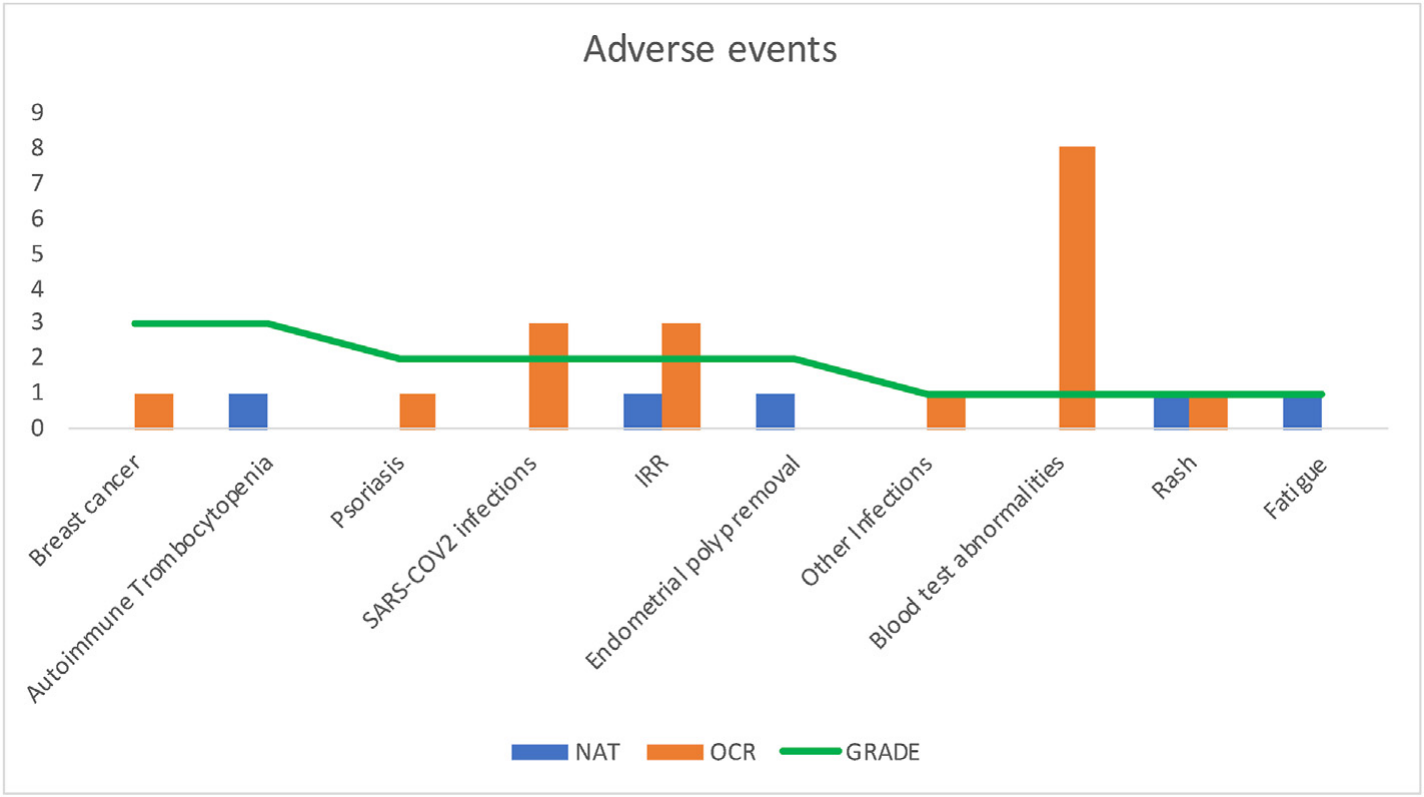
**Adverse events in the matched cohort.** Adverse events are reported in x-axis from the most severe (left) to the less severe (right). Grading of adverse events according to the CTCAE-5 is represented by the level of the green line intersection on y-axis. Number of patients experiencing the event are reported in the y axis. Natalizumab-treated patients are represented in blue, Ocrelizumab-treated patients in orange. IRR: infusion-related reaction.

Safety analysis was conducted in the matched cohort to avoid hypothetical bias due to baseline demographics characteristics or different follow-up times, possibly affecting AEs. Out of 140 patients, only 19 developed AEs (4 and 15 respectively from NTZ and OCR group). Patients treated with OCR had a higher risk of developing AEs compared to NTZ group (OR 4.50 (1.53–16.50, p ​= ​0.011). Seven out of 15 patients treated with OCR and experiencing AEs were part of the RCTs cohort. Five and 18 AEs were reported respectively in 4 NTZ-treated patients vs 15 OCR-treated patients. None of these events was considered life-threatening. Adverse events reported were infusion-related reactions (IRR), SARS-COV2 infection requiring treatment or hospitalization and other infections, abnormalities in blood test (anemia, lymphocytopenia, hypogammaglobulinemia and others), tumors (breast cancer) development of autoimmune diseases (psoriasis, autoimmune thrombocytopenia), minor surgeries (endometrial polyp removal) and others. Frequencies and grading of AEs are reported in Fig. 4.

### Treatment persistence analysis

Twenty-one patients treated with NTZ discontinued the treatment because of JCV positivity (n ​= ​15), AEs (n ​= ​1), EDSS progression (n ​= ​1), pregnancies (n ​= ​2) or they were lost to follow-up (n ​= ​1) or transferred to other centers (n ​= ​1) (Table 2). Among them, patients discontinuing because of JCV+, EDSS progression or AEs (n ​= ​17) switched to another DMT. After discontinuing NTZ, 12 patients were commenced on Cladribine, 2 Fingolimod, 3 anti-cd20 monoclonal antibodies.

**Table 2:** Reasons for treatment discontinuation.

	NTZ, n ​= ​21	OCR, n ​= ​14
JCV positivity	15	0
Adverse events	1	1
Pregnancy	2	1
Transferred to another MS center	1	8
Lost at follow-up	1	1
Patient decision	0	1
EDSS progression	1	2

EDSS: Expanded disability status scale, JCV: John Cunningham Virus, NTZ: natalizumab, OCR: ocrelizumab.

Fourteen patients treated with OCR discontinued the treatment because of pregnancy (n ​= ​1), patient decision (n ​= ​1), adverse events (n ​= ​1), EDSS progression (n ​= ​2) or they were lost-to follow-up (n ​= ​1) or transferred to another MS center (n ​= ​8). Among patients followed-up in our centers, the 2 patients switching treatment because of EDSS progression started treatment with Ofatumumab (n ​= ​1) and Mitoxantrone (n ​= ​1).

No differences in treatment discontinuation was found between the 2 cohorts [OR 0.58 (0.26–1.26) p ​= ​0.174].

When performing a sensitivity analysis excluding patients transferred to other MS centers (n ​= ​1 for NTZ, n ​= ​8 for OCR), patients treated with NTZ had a higher probability of treatment discontinuation [OR ​= ​0.26 (0.09–0.67, p ​= ​0.008)] than patients treated with OCR.

## Discussion

In this study, we analyzed the comparative effectiveness, safety profile, and treatment persistence of two highly effective DMTs, NTZ and OCR, over a 5-year follow-up period in a mixed cohort of relapsing and progressive MS patients.

In our effectiveness analysis, we found no significant differences between the NTZ and OCR cohorts in achieving no evidence of disease activity (NEDA-3) or in reducing relapses, MRI activity, and EDSS progression. A previous real-world study comparing OCR vs NAT in RRMS over 30 months also found no differences in NEDA-3 in 55 matched pairs, even though OCR seemed superior to NAT in preventing relapse occurrence [21]. In our cohort, we observed no significant differences in time to first relapse at 1, 3 and 5 years of FU. Notably, the German OCR cohort had over 40 ​% of patients previously treated with NTZ, potentially affecting the efficacy results, whereas in our study, patients switching between NTZ and OCR were excluded to avoid such confounding factors.

Another real-world study comparing the effectiveness of Fingolimod, Natalizumab and Ocrelizumab in RRMS found no differences in 310 NTZ vs OCR matched-pairs in annualized relapse rate(ARR), confirmed disability accumulation, proportion of relapse-free and MRI-activity-free patients over a relatively short follow-up (FU) (2 years) [22]. Secondary analyses, performed excluding patients who had previously received one of the two therapies, showed comparable ARR at year 1 and over 2 years, consistent with our findings. However, among patients discontinuing fingolimod, OCR reduced ARR by 33 ​% and lowered the cumulative hazard of time to the first relapse by 43 ​% compared to NTZ at 2 years, though no difference was found in the risk of disability accumulation [23].

A recent French study further demonstrated comparative effectiveness of NTZ versus anti-CD20 monoclonal antibodies (rituximab and ocrelizumab) in highly active RRMS after fingolimod discontinuation, when evaluating time to first relapse and relapse rates, EDSS progression, and MRI lesion dynamics over 2 years using multidimensional penalized splines [24]. Our study provides a longer follow-up compared to previous studies, thus confirming the comparative effectiveness of the two drugs over a longer observation period. Notably, the only recent study with a 5-year follow-up which compared OCR-treated patients in OPERA I and II with different DMTs in a real-world cohort, found that OCR reduced the risk of relapse by 46 ​% compared to NTZ, though its effect on disability accumulation was only marginally significant (p ​= ​0.048) [25].

In our study, we evaluated a mixed cohort of relapsing-remitting, secondary progressive and primary progressive MS patients. In clinical practice monoclonal antibodies like NTZ and OCR are often prescribed as a highly effective treatments not only to reduce early inflammatory activity but also to prevent progression independent of relapse activity, both in treatment-naïve patients and those switching from other DMTs. Most real-world studies have focused on RRMS, especially in treatment-naïve patients, to minimize confounding factors. For instance, a recent Italian study involving highly active, treatment-naïve RRMS patients found no differences in NEDA-3 between OCR and NTZ at 3 years [26]. A registry-based study of 195 treatment-naïve RRMS pairs with a 1.6-year follow-up also reported no significant differences between NTZ and OCR in progression independent of relapse activity (PIRA) or in reaching irreversible disability milestones (EDSS scores of 4 or 6) [27]. Our study extends these findings to patients transitioning from other DMTs and provides insights into progressive MS (PMS) patients, who constitute 20 ​% of the total population analyzed, thereby reflecting a more typical clinical population.

In our safety analysis, patients treated with OCR had a higher risk of AEs compared to those on NTZ, particularly regarding blood test abnormalities and infections. The B-cell-depleting mechanism of OCR likely contributes to the increased susceptibility to infections, in contrast to NTZ's immune cell sequestration in the peripheral circulation. Previous studies identified nasopharyngitis, urinary tract infections, and infusion-related reactions as the most common AEs associated with ocrelizumab, affecting approximately 10 ​% of patients [28,29]. However, no life-threatening events were observed in either cohorts.

In terms of treatment persistence, we found no significant difference in discontinuation rates between the NTZ and OCR cohorts. After excluding patients transferred to other MS centers, OCR showed a higher treatment persistence than NTZ. However, it is important to acknowledge that we cannot confirm whether all patients who transferred to other centers continued their previous treatment (e.g., OCR), which limits the interpretability of this analysis. Most NTZ-treated patients discontinued therapy due to safety concerns, particularly JCV positivity. This finding aligns with previous studies that identify JCV positivity or increased antibody index levels as the leading cause of premature NTZ discontinuation [21,26]. Indeed, lack of effectiveness seems to play a marginal role in NTZ discontinuation, with a more common treatment strategy of de-escalation toward less efficacious DMTs [25]. In our cohort, patients discontinuing NTZ were primarily switched to cladribine or anti-cd20 monoclonal antibodies or fingolimod. On the other hand, OCR-treated patients were more frequently transferred to other MS centers than NTZ-treated patients, though this affected only a small proportion of the total cohort. High persistence and adherence to OCR over 5 years have been recently reported, with few cases of discontinuation due to lack of efficacy or AEs [30].

This study has some limitations. The observational retrospective design, together with the inclusion of both patients from the real-world and RCTs, could have resulted in a bias in data collection, especially in terms of side effects. MRI data were not available for all the patients in the cohort, which may affect the accuracy of the results. Moreover, re-baseline MRI was not routinely performed: however, MRI activity occurred within 4 weeks of treatment was excluded in the effectiveness analysis. Our sample size after propensity-score matching was relatively small, preventing us from separately evaluating the efficacy of the two drugs in patients transitioning from low-versus high-efficacy treatments. Additionally, our findings may not be generalizable to populations with different demographic or clinical characteristics, limiting their applicability to other clinical contexts.

In conclusion, our study demonstrates comparable effectiveness between NTZ and OCR in terms of relapse rates, MRI activity, and EDSS progression. In comparison to NTZ, OCR was associated with a higher incidence of mild to moderate adverse events, and higher to comparable treatment persistence.

## Data availability

The datasets are available from the corresponding author on reasonable request.

## Disclosures

Antonio Carotenuto disclosed research grants from ECTRIMS-MAGNIMS and Almirall, travel/meeting expenses from Novartis, Janssen, Roche and Merck and speaking honoraria from Merck, BMS, Biogen, Novartis, Roche and Almirall. Marcello Moccia has received research grants from MUR PNRR Extended Partnership (MNESYS no. PE00000006, and DHEAL-COM no. PNC-E3-2022-23683267), ECTRIMS- MAGNIMS, UK MS Society, and Merck; honoraria from Biogen, BMS Celgene, Janssen, Merck, Roche, and Sanofi- Genzyme; and salary as Editorial Board Member from Neurology (AAN, MN, US) and Multiple Sclerosis Journal (Sage, UK). Serena Ruggieri has received honoraria from Biogen, BMS, Merck, Novartis, BMS, Roche for consulting services, speaking and/or travel support. Giovanna Borriello received fee for advisory from Almirall, Biogen, BMS, Merck, Novartis, Roche, Sanofi. Roberta Lanzillo has received honoraria from Biogen, Merck, Novartis, Roche and Teva. Vincenzo Brescia Morra has received research grants from Italian MS Federation and Roche; and honoraria from Almirall, Biogen, BMS Celgene, Janssen, Merck, Novartis, Roche, Sanofi-Genzyme, Bayer, Mylan, Teva and Viatris. Carlo Pozzilli has served on scientific advisory boards for Novartis, Merck, Biogen, Sanofi, Genzyme, Teva, and Actelion; received funding for travel and speaker honoraria from Biogen, Teva, Sanofi Genzyme, Actelion, and Novartis; received research support from Biogen, Teva, Novartis, and Genzyme. Maria Petracca discloses travel/meeting expenses from Novartis, Janssen, Roche and Merck; speaking honoraria from HEALTH&LIFE S.r.l., AIM Education S.r.l., Biogen, Novartis and FARECOMUNICAZIONE E20; honoraria for consulting services and advisory board participation from Biogen; research grants from Baroni Foundation and the Italian Ministry of University and Research. Elena Barbuti, Alessia Castiello, Valeria Pozzilli, Ilaria Tomasso have nothing to disclose.

## Author contributions

**Elena Barbuti:** conceptualization, methodology, formal analysis, investigation, data curation, writing – original draft, writing – review and editing, visualization. **Alessia Castiello**: investigation, data curation, writing – review and editing. **Valeria Pozzilli:** investigation, data curation, writing – review and editing. **Antonio Carotenuto**: investigation, data curation, writing – review and editing. **Ilaria Tomasso:** investigation, data curation, writing – review and editing. **Marcello Moccia:** investigation, data curation, writing – review and editing. **Serena Ruggieri** investigation, data curation, writing – review and editing. **Giovanna Borriello:** investigation, data curation, writing – review and editing. **Roberta Lanzillo:** investigation, data curation, writing – review and editing. **Vincenzo Brescia Morra;** investigation, data curation, writing – review and editing. **Carlo Pozzilli:** conceptualization, methodology, data curation, investigation, writing – review and editing. Supervision. **Maria Petracca**: conceptualization, methodology, formal analysis, writing – original draft, writing – review and editing, supervision.

## Funding

This research received no specific grant from any funding agency in the public, commercial, or not-for-profit sectors.

## Declaration of competing interest

The authors declare the following financial interests/personal relationships which may be considered as potential competing interests. Many authors (see manuscript) report a relationship with Roche, Teva, Janssen, Merck, Biogen, Novartis, Mylan, Viatris, Bayer, Sanofi, Genzyme, BMS, Bristol, Almirall, Celgene, Actelion, CSL, Behring, HEALTH&LIFE, AIM Education, FARECOMUNICAZIONE E20 that includes: board membership, consulting or advisory, funding grants, speaking and lecture fees, and travel reimbursement.
